# Genome-Wide Analysis of Glucocorticoid Receptor Binding Regions in Adipocytes Reveal Gene Network Involved in Triglyceride Homeostasis

**DOI:** 10.1371/journal.pone.0015188

**Published:** 2010-12-20

**Authors:** Chi-Yi Yu, Oleg Mayba, Joyce V. Lee, Joanna Tran, Charlie Harris, Terence P. Speed, Jen-Chywan Wang

**Affiliations:** 1 Department of Nutritional Science & Toxicology, University of California, Berkeley, California, United States of America; 2 Department of Statistics, University of California, Berkeley, California, United States of America; 3 Department of Medicine, Gladstone Institute of Cardiovascular Disease, Cardiovascular Research Institute, University of California San Francisco, San Francisco, California, United States of America; National Institute on Aging, National Institutes of Health, United States of America

## Abstract

Glucocorticoids play important roles in the regulation of distinct aspects of adipocyte biology. Excess glucocorticoids in adipocytes are associated with metabolic disorders, including central obesity, insulin resistance and dyslipidemia. To understand the mechanisms underlying the glucocorticoid action in adipocytes, we used chromatin immunoprecipitation sequencing to isolate genome-wide glucocorticoid receptor (GR) binding regions (GBRs) in 3T3-L1 adipocytes. Furthermore, gene expression analyses were used to identify genes that were regulated by glucocorticoids. Overall, 274 glucocorticoid-regulated genes contain or locate nearby GBR. We found that many GBRs were located in or nearby genes involved in triglyceride (TG) synthesis (*Scd-1*, *2*, *3*, *GPAT3*, *GPAT4*, *Agpat2*, *Lpin1*), lipolysis (*Lipe*, *Mgll*), lipid transport (*Cd36*, *Lrp-1*, *Vldlr*, *Slc27a2*) and storage (*S3-12*). Gene expression analysis showed that except for *Scd-3*, the other 13 genes were induced in mouse inguinal fat upon 4-day glucocorticoid treatment. Reporter gene assays showed that except *Agpat2*, the other 12 glucocorticoid-regulated genes contain at least one GBR that can mediate hormone response. In agreement with the fact that glucocorticoids activated genes in both TG biosynthetic and lipolytic pathways, we confirmed that 4-day glucocorticoid treatment increased TG synthesis and lipolysis concomitantly in inguinal fat. Notably, we found that 9 of these 12 genes were induced in transgenic mice that have constant elevated plasma glucocorticoid levels. These results suggested that a similar mechanism was used to regulate TG homeostasis during chronic glucocorticoid treatment. In summary, our studies have identified molecular components in a glucocorticoid-controlled gene network involved in the regulation of TG homeostasis in adipocytes. Understanding the regulation of this gene network should provide important insight for future therapeutic developments for metabolic diseases.

## Introduction

Glucocorticoids regulate many facets of energy metabolism. Excessive glucocorticoid exposure, whether exogenous or endogenous, is associated with the development of metabolic syndrome, a constellation of metabolic risk factors that include insulin resistance, central obesity, dyslipidemia and hypertension [1], [2]. Animal model studies, either applying genetic [3], [4] or pharmacological approaches [5], [6], [7], have shown that reducing the glucocorticoid signaling in vivo protects the animals from various metabolic disorders and improves their metabolic profiles. Intriguingly, several studies have shown that increasing active glucocorticoid levels specifically in adipocytes can cause metabolic syndrome [8], [9]. Conversely, transgenic mice that have decreased levels of active glucocorticoids in adipocytes are protected from diet-induced obesity and have improved glucose tolerance and insulin sensitivity [10]. Thus, these data highlight the critical role of adipocytes in glucocorticoid-regulated energy homeostasis and demonstrate that modulating glucocorticoid signaling in this cell type exclusively can have significant impacts on whole body metabolism.

In adipocytes, glucocorticoids modulate both glucose and lipid homeostasis. For the former, glucocorticoids inhibit insulin-stimulated glucose uptake [11], [12], [13]. For the latter, depending on nutritional state, glucocorticoids exert distinct effects on lipid metabolism, increasing lipogenesis in the fed state [14], [15] and increasing lipolysis in the fasted state [16], [17], [18]. Furthermore, glucocorticoids promote re-distribution of body fat [19]. Patients of Cushing's syndrome, which is characterized by high plasma cortisol levels, have fat accumulation in the depots of the abdomen, the nape of the neck and the cheeks, but reduced fat storage in other subcutaneous depots [20]. Overall, the effects of glucocorticoids on lipid metabolism could be affected by other environmental cue and are also region specific. It is important to note that while the glucocorticoid-regulated metabolic phenotypes are well described, the mechanisms underlying these metabolic effects are not entirely elucidated. Glucocorticoids exert their biological functions through an intracellular glucocorticoid receptor (GR). GR is a transcriptional regulator, which upon binding to cognate ligands, occupies specific genomic GREs, and modulates the transcription of nearby genes [21]. The products of these “primary” target genes either directly affect cellular physiology or modulate the expression of other sets of genes (“secondary” target genes), which then confer hormonal responses [22]. Identifying primary target genes that initiate physiological responses will shed new light on the mechanisms governing glucocorticoid actions. In different cell types glucocorticoids affect distinct physiological processes by inducing specific gene expression program. Notably, very few GR primary target genes in adipocytes have been identified to date.

To identify GR primary target genes in adipocytes, in this report, we applied a combination of chromatin immunoprecipitation (ChIP) and a high throughput DNA sequencing technique (called ChIPseq) to isolate genome-wide GR binding regions (GBRs) in 3T3-L1 adipocytes. Intriguingly, we found that many genes involved in TG homeostasis, including those in TG biosynthetic and lipolytic pathways, contain or are located near GBRs. We further investigated the regulation of these genes by glucocorticoids in mouse adipose tissue. Moreover, to further confirm that these genes are GR primary targets, GBRs from distinct genes were inserted into a reporter plasmid to determine whether they can mediate a glucocorticoid response. Finally, to learn whether the glucocorticoid-induced gene expression change translates to corresponding physiological outputs, we applied stable isotope labeling technique to monitor the effect of glucocorticoids on TG metabolism in vivo.

## Methods

### Cell culture

The 3T3-L1 (Mouse embryonic fibroblast-adipose like cell line) cells, kindly provided by Dr. Hei Sook Sul (UC Berkeley), were maintained in Dulbecco's modified Eagle's medium (DMEM; Mediatech) containing 10% fetal bovine serum (FBS; Tissue Culture Biological) and incubated at 37°C with 5% CO_2_. 3T3-L1 cells at 2 days post-confluence were differentiated into adipocytes using differentiation-inducing medium (1 µM dexamethasone (DEX), 0.5 mM methylisobutylxanthine, and 1.67 µM insulin in DMEM with 10% FBS). The 3T3-L1 cells were maintained in differentiation media for 3 days. The cells were then returned to medium consisting of 10% FBS in DMEM without drugsfor an additional 3 days. Before embarking on a ChIP-seq experiment, differentiated 3T3-L1 adipocytes were washed with 1× phosphate-buffered saline (1× PBS buffer) and cultured in DMEM supplemented with 5% stripped FBS (J.R. Scientific) for 24 hours. 3T3-L1 adipocytes were then exposed to 500 nM dexamethasone (DEX) or an equal volume (0.05% v/v of media) of ethanol as a vehicle control for 1 h at 37°C in DMEM with 5% stripped FBS.

### Animals

Male 8-week-old C57BL/6 mice were purchased from Charles River. Animals were injected daily with 5 mg/kg DEX (Sigma) or PBS for 4 days. After 24 hours of the last injection, animals were sacrificed for various assays. Transgenic mice overexpressing corticotropin-releasing hormone (CRH) were provided by Mary Stenzel-Poore [23]. The Office of Laboratory Animal Care at the University of California, Berkeley (#R306-0111) approved all animal experiments conducted in this paper.

### ChIPseq

After 3T3-1L adipocytes were treated, DEX- or ethanol-treated cells (two 15 cm plates for each treatment) were cross-linked in 1% formaldehyde for 10 min at room temperature, followed by quenching of the cross-linking reaction in 0.125 M glycine for 5 min. After washing the cells with 1× PBS, they were lysed and scraped in cell lysis buffer (50 mM HEPES-KOH at pH 7.4, 1 mM EDTA, 150 mM NaCl, 10% glycerol, 0.5% Triton X-100) added with protease inhibitor cocktails (Calbiochem). The cell lysate was incubated for 10 min at 4°C and the crude nuclear extract was collected by centrifugation at 600×g for 5 min at 4°C. We resuspended nuclei in 1 mL of ice-cold RIPA buffer (10 mM Tris-HCL at pH 8.0, 1 mM EDTA, 150 mM NaCl, 5% glycerol, 1% Triton X-100, 0.1% sodium deoxycholate, 0.1% SDS, supplemented with protease inhibitor) and fragmented chromatin to 200–500 bp with eleven pulses of 20 sec at 30% power with a Branson Sonifier 250 sonicator at 4°C. To remove insoluble components, we centrifuged the samples at 13,000 rpm for 15 min at 4°C and recovered the supernatant. The supernatant was then added to 1 µg of rabbit polyclonal anti-GR (N499) antibody to immunoprecipitate GR-bound chromatin at 4°C overnight. We next added 100u of 50% protein A/G plus-agarose bead slurry (Santa Cruz Biotechnology) into each immunoprecipitation and incubated it for 2 h at 4°C. After incubation, we washed the beads five times with RIPA buffer containing 510 mM NaCl, followed by five times with LiCl buffer (20 mM Tris at pH 8.0, 1 mM EDTA, 250 mM LiCl, 0.5% NP-40, 0.5% sodiumdeoxycholate, supplemented with protease inhibitor) and five times with RIPA buffer containing 510 mM NaCl. After removing the remaining wash buffer, each IP reaction was added to 75 µl of proteinase K solution (TE pH 8.0, 0.7% SDS, 200 µg/ml proteinase K) and incubated for 3 h at 55°C, followed by incubating overnight at 65°C to reverse formaldehyde cross-links. We further purified the DNA with a QIAquick PCR purification kit (Qiagen), eluting in 30 µl of Qiagen Elution Buffer.

For each ChIPseq experiment, we followed the procedures of Illumina genomic DNA library preparation for sequencing. In brief, ChIP DNA fragments were blunted and ligated to sequencing adapters. The adapter-ligated DNA was amplified with 25 rounds of PCR. We electrophoresed the amplified products on a 2% agarose gel. We excised a region of gel containing DNA fragments 150–300 bp in length and extracted DNA from the gel slice with a Qiagen Gel Extraction kit. We then ensured the DNA quality by Bioanalyzer (Agilent). The resulting DNA library was sequenced on the Illumina Genome Analyzer (Vincent J. Coates Genomic Sequencing Laboratory, UC Berkeley). The reads that have passed Illumina's internal quality filter were retained for further analysis. Reads were aligned to mm9 assembly of mouse genome by Illumina's default aligner ELAND (allowing up to 2 mismatches in first 32 bps). The aligned reads were given as input to peak-finding software Model-based Analysis of ChIPseq (MACS). The default p-value cutoff criterion was at 10^−5^ (the effective genome size was set to 2.2 Gb). The underlying DNA sequences of the identified peak regions were obtained with a python script and further analyzed with web-based motif-finding tools.

### Annotation of genes

The distribution of GR-binding sites, relative to nearest genes, was determined with PinkThing (http://pinkthing.cmbi.ru.nl). All sequences associated with the peaks were obtained from the *Mus musculus* NCBI m37 genome assembly (mm9; July 2007).

### Motif search and gene ontology analysis

BioProspector [24] was used to search for 14- or 8-bp motifs in the ChIPseq data. The top 10–20 scoring output motifs from BioProspector were then compared to known binding sites in TRANSFAC V11.3 database using STAMP [25]. A motif discovery program, cis-regulatory element annotation system (CEAS) (http://ceas.cbi.pku.edu.cn) (Ji et al. 2006), was also performed to obtain the enriched transcription factor motifs located in ChIPseq-identified GBRs.

The Database for Annotation, Visualization and Integrated Discovery (DAVID) (http://david.abcc.ncifcrf.gov/home.jsp) was used to perform gene ontology analysis. Below are the list of categories selected for analysis: Disease: OMIM_DISEASE, Functional Categories: COG_ONTOLOGY, SP_PIR_KEYWORDS, UP_SEQ_FEATURE, Gene_Ontology: GOTERM_BP_ALL, GOTERM_CC_ALL, GOTERM_MF_ALL, PANTHER_BP_ALL, PANTHER_MF_ALL, Pathway: BBID, BIOCARTA, KEGG_PATHWAY.

### RNA isolation and quantitative PCR

Total RNA was isolated from mouse inguinal fat using TRI Reagent® RT (Molecular Research Center, Inc.). To synthesize random-primed cDNA, 0.5 µg of total RNA, 4 µl of 2.5 mM dNTP, and 2 µl of random primers (New England Biolabs) were mixed at a volume of 16 µl and incubated at 70°C for 10 min. Then, a 4-µl cocktail containing 25 units of Moloney Murine Leukemia Virus (M-MuLV) Reverse Transcriptase (New England Biolabs), 10 units of RNasin (Promega), and 2 µl of 10× reaction buffer (New England Biolabs) was added and incubated at 42°C for 1 h. The reaction was then incubated at 95°C for 5 min. The resultant cDNA was diluted to 200 µl, and 3.5 µl was used to perform qPCR using EVA QPCR SuperMix Kit (Biochain) per manufacturer's protocol. qPCR was performed in either a 7900HT, 7500HT or StepOne PCR System (Applied Biosystems) and analyzed by using the ΔΔ-Ct method as supplied by the manufacturer (Applied Biosystems). *Rpl19* gene expression was used for internal normalization. Primer sequences are listed in Table S2.

### Plasmids, transfection, and luciferase reporter assay

pGL4.10-E4TATA reporter plasmid was generated by insertion of a 50-bp minimal *E4* TATA promoter sequence (Lin et al. 1988) into the *Bgl* II to *Hind* III sites of vector pGL4.10 to drive luciferase expression (Bolton et al. 2007). Each chosen GBR fragment, extending 100–150 bp upstream and downstream, was amplified from genomic 3T3-L1 DNA (primer sequences are listed in Table S2) using Phusion High–Fidelity DNA Polymerase (New England BioLabs) and cloned into the pGL4.10-E4TATA vector by either *Xho* I/*EcoR* V or *Kpn* I/*Xho* I sites. SuperFect transfection reagent (Qiagen) was used to transfect 3T3-L1 preadipocytes in 12-well plates according to the technical manual. Twenty-four hours post-transfection, cells were treated with either 1 µM DEX or ethanol, in DMEM with 5% stripped FBS for 24 hours. Cells were then harvested, and luciferase activity was measured with a Dual-Luciferase Reporter Assay kit (Promega), following the technical manual.

### Measurement of triglyceride concentration and synthesis by stable isotope

Mice were administered by an intraperitoneal injection of heavy water (^2^H_2_O)(0.035 ml/g body weight of 100% atomic percentage excess ^2^H_2_O), and they were maintained on 8% ^2^H-labeled drinking water for 7 days. After 3 days of giving 8% ^2^H-labeled drinking water, mice were treated with DEX (5 mg/kg body weight) or PBS for 4 consecutive days. The mice were sacrificed, and inguinal fats were collected to measure the rate of TG synthesis as previously described (Bederman et al. 2009). The TG composition of inguinal fats was studied using a thin layer chromatography (TLC) method, which begins with briefly homogenizing inguinal fat samples in Tris Sucrose buffer (50 mM Tris-HCl at pH 7.4, 250 mM sucrose, supplemented with protease inhibitors). Lipids were extracted using chloroform∶methanol (2∶1) and separated on Whatman* Adsorption 60Å Silica Gel TLC Plates with the solvent hexane∶ethyl ether∶acetic acid (v∶v∶v80∶20∶1). TLC plates were exposed to iodine vapor in order to visualize the TG bands. The TG bands were scraped and added to 1 ml of a 1∶1 mixture of the 8% ethanolic NaOH and 4% hydroxylamine solution. The TG reaction was thoroughly vortexed and incubated at 65°C for 2 minutes. The TG samples were cooled for 5 minutes at room temperature and 2.5 ml of fresh ferric perchlorate reagent was added. Next, 250 µl of each TG sample was loaded in a 96 well ELISA plate with a serial dilution of 250 mM Triolein (Sigma) as a set of standards. The plate was read at 530 nm after 30 minutes. The TG levels were calculated and expressed as mmol per mg tissue weight. To make the fresh ferric perchlorate reagent, 5 g of ferric perchlorate was dissolved in 10 ml of 70% HClO_4_ and 10 ml of water. This solution was diluted with 80 ml cold 100% ethanol to make a stock ferric perchlorate solution. 4 ml of the stock ferric perchlorate solution was then combined with 3 ml of 70% HClO_4_ and 93 ml cold 95% ethanol.

### Lipolysis Assay

The lipolysis assay was conducted as previously described (Jaworski et al. 2009). Explants from freshly removed inguinal fat pads (∼100mg) were incubated at 37°C in 500uL Krebs-Ringer Buffer (12mM HEPES,121mM NaCl, 4.9mM KCl, 1.2 mM MgSO4 and 0.33mM CaCl) with 3% BSA and 3mM glucose. Glycerol release was determined over time using free glycerol reagent (Sigma). Measurements were normalized to total protein content of the adipose sample using Bradford protein dye (BioRad).

### Microarrays and Data Analysis

3T3-L1 adipocytes were cultured in DMEM supplemented with 5% stripped FBS (J.R. Research) for 24 hours and then treated with 500 nM DEX or an equal volume (0.05% v/v of media) of ethanol as a vehicle control for 6 hours at 37°C in DMEM with 5% stripped FBS. Total cellular RNA was isolated utilizing the NucleoSpin RNA II kit (Macherey-Nagel). RNA isolates were first quantified by standard spectrophotometry, and then qualitatively evaluated by capillary electrophoresis employing the Bio-Rad Experion system per the manufacturer's instruction. Biotin-labeled cRNA samples were prepared with 750 ng of total RNA template. Following synthesis and purification, the biotin-labeled samples were evaluated by both 260/280 absorbance spectrophotometry and capillary electrophoresis. The final labeled cRNA samples were hybridized overnight against 48,000 transcripts using MouseWG-6 BeadChip arrays (Illumina, San Diego, CA). The Illumina microarrays were processed at the UCSF Genomics Core. All treatments were done in triplicates, and the same batch of microarrays was used for all treatments. The Illumina expression arrays were pre-processed using lumi package [26]. The differential expression analysis was performed using the Limma package [27]. These packages are all available in R/BioConductor. Probes were selected for further analysis if the fold-induction was greater than 2 and multiple testing adjusted the p-value to less than 0.05 using Benjamini and Hochberg procedure (BH-adjusted p-value) [28]. The heatmaps of log intensities of genes across different experiments were produced using Cluster and TreeView software [29]. The microarray data is available at the Gene Expression Omnibus Web site (http://www.ncbi.nlm.nih.gov/geo/) under accession No. GSE24105.

## Results

### Identification of genome-wide GR binding regions in 3T3-L1 adipocytes

**Figure 1:**
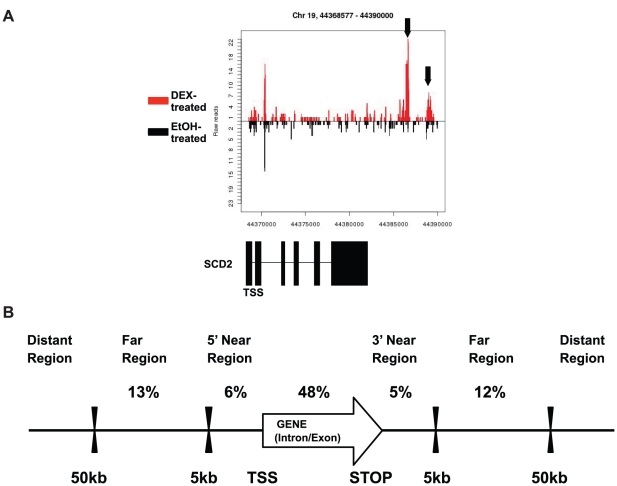
Genomic distribution of glucocorticoid receptor binding sites binding sites and response elements. A) An example of ChIPseq data showing the number of ChIPseq sequencing tag in *Scd-2* gene. ChIP-seq data are plotted as the density of 36-bp tags mapping to the region. The density is separated to show the reads (Y-axis) mapping to the reference genome (X-axis) in DEX (red) or ethanol (black) treated samples. B) Enrichment of GBRs in genomic features. The percentage of all identified GBRs in each type of region was shown.

We used ChIPseq to identify the genomic locations bound by mouse GR in 3T3-L1 adipocytes in response to the treatment of a synthetic glucocorticoid, dexamethasone (DEX). We sequenced the ChIP DNA with an Illumina Genome Analyzer and aligned the sequence reads to the mouse genome using Illumina's ELAND program. Model-based Analysis of ChIPseq (MACS) algorithm was then used to identify enriched genomic regions in DEX-treated samples vs. ethanol-treated samples. When p value<10^−5^ was used, we identified 8,848 genomic positions occupied by GR upon DEX treatment. We used PinkThing (http://pinkthing.cmbi.ru.nl) to assign GBRs to mouse genes based on proximity, and target sites were grouped depending on their position relative to the nearest gene. Fig. 1A is an example of these analyses. In chromosome 19, downstream of the *Scd-2* gene, we identified two GBRs, as the number of sequencing reads at these two regions were significantly enriched in DEX-treated samples than in ethanol-treated samples (Fig. 1A). A complete list of binding sites at all significant thresholds is available in Dataset S1. Overall, GR preferentially bound in the intron regions (48%, Fig. 1B). 13% of GBRs were located between 5–50 kb 5′ of transcription start site (TSS), whereas 17% were located within 50 kb from 3′ of stop codon (Fig. 1B). Interestingly, only 6% of GBRs were located inside of 5 kb from TSS (Fig. 1B). Furthermore, 16% of GBRs were located 50 kb upstream of TSS or 50 kb downstream of the stop codon (Fig. 1B). Other studies using ChIPseq or ChIP on chip to isolate binding sites for nuclear receptors also showed that only approximately 10% of binding sites are located inside of 5 kb from the TSS, whereas the majority of binding sites were located in the intron and/or far distant region [30], [31], [32], [33], [34], [35].

### Identifying GR-regulated Genes in 3T3-L1 Adipocytes using microarray

Potential GR primary target genes that contain or are located nearby GBRs should have expression regulated by glucocorticoids. To identify potential GR primary target genes in adipocytes, we performed microarray analyses to isolate genes whose expression is modulated by glucocorticoids upon 6-hour-DEX treatment in 3T3-L1 adipocytes. We found that 421 genes were significantly induced by DEX by more than 1.5 fold. Among these genes, 292 genes contain GBRs in their genomic regions, or GBRs located within 100 kb upstream of their TSS or within 100 kb downstream of their stop codon (Table 1). In contrast, 198 genes were inhibited by DEX by more than 1.5 fold. Among these genes, 45 genes contain GBRs in their genomic regions, or GBRs are located within 100 kb upstream of their TSS or within 100 kb downstream of their stop codon (Table 1). All of the 620 DEX-regulated genes identified by microarray are shown in Dataset S2. Overall, we identified 337 glucocorticoid responsive genes (Dataset S3), which had their expressions modulated, up or down, by glucocorticoids in 3T3-L1 adipocytes, and they contain or are located nearby GBRs.

**Table 1:** Number of Genes Regulated by Glucocorticoids and containing GBRs in 3T3-L1 Adipocytes.

	Glucocorticoid-regulated genes	Glucocorticoid-regulated genes containing GBRs*	% of Glucocorticoid regulated genes containing GBRs*
**Activated**	421	292	59%
**Repressed**	198	45	23%
**Total**	619	337	54%

*GBRs are located in the intron or within 100kb upstream from the TSS or 100kb downstream from the stop codon.

We used a web-based gene ontology tool, DAVID, to classify these 337 glucocorticoid responsive genes. The top categories of genes identified in the functional annotation chart include those involved in the regulation of distinct developmental processes, such as blood vessel and mammary gland formation, and the regulation of apoptosis, cell proliferation and signal transduction (Dataset S4). Moreover, genes involved in lipid metabolism are also significantly presented (Dataset S4). The genes identified in each category of gene ontology are shown in Dataset S4. Notably, among these 337 genes, *Dusp1* [36], *Insig2* [37], *Lcn2* [38], *Pik3r1* [39], and *Ptgds* [40] have been previously shown to regulate the insulin action in adipocytes. The overexpression of *Dusp1*, *Insig2*, and *Lcn2* has been linked to the development of insulin resistance. Thus, the induction of these three genes by glucocorticoids is consistent with their repressive effect on insulin-stimulated glucose uptake. Conversely, *Ptgds* play a positive role in the regulation of insulin-stimulated glucose uptake. Moreover, several potential GR primary targets are previously shown to be involved in the adipogenesis: *Ctsl* [41], *Dusp1* [42], *Insig2* [43], *Lpin1* [44], *Ptgds* [45], *Rgs2* [46], *Scd2* [47], *Sgk1* [48]. *Tsc22d3*, however, was found to inhibit the adipocyte differentiation [49]. Finally, some potential target genes are the components of the signaling pathways regulating adipogenesis, such as TGFß (*Smad3*, *FST*, and *Lox*) [50] and wnt (*FZD1*) [51].

**Figure 2:**
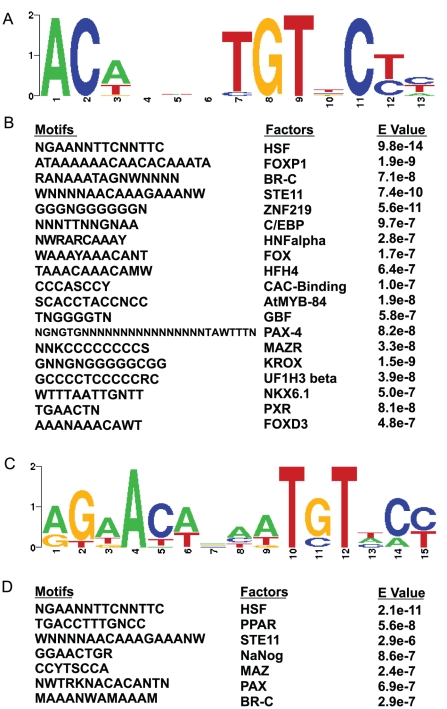
GREs and other *cis*-regulatory motifs are enriched within GBRs. Unbiased motif search for GBRs in or nearby glucocorticoid-activated and –repressed genes. A) Bioprospector analysis identified a motif that is highly similar to a consensus GRE in STAMP for glucocorticoid-induced genes. B) Bioprospector and STAMP identified putative motifs for other transcription factors in GBRs of glucocorticoid-activated genes. Top 15 motifs other than GRE were shown. C) Bioprospector analysis identified a motif that matches a consensus ARE in STAMP in glucocorticoid-repressed genes. Notably, the sequences of this consensus ARE is very similar to those of consensus GRE. D) Bioprospector and STAMP identified putative motifs for other transcription factors in GBRs of glucocorticoid-repressed genes. Top 15 motifs other than ARE/GRE were shown.

We performed a combination of Bioprospector [24] and STAMP [25] to search for consensus motifs within GBRs located in or nearby genes that were induced or repressed by glucocorticoids. In Bioprospector, either a width of 14 or 8 was used for the analyses. For glucocorticoid-activated genes, a motif for GRE was highly represented from these analyses (Fig. 2A). The motif for GRE is usually similar to that of ARE (androgen response element). Interestingly, these the analysis suggested that heat shock factor (HSF) could also bind to this similar motif. Furthermore, the rest of the binding motifs other than GRE include FOXP1, STE11, HFH4, FOX, FOXD3, UF1H3beta, BR-C, ZNF219, E4BP4, C/EBP, HNF3alpha (aka FOXA1), CAC-binding, AtMYB-84, GBF, PAX-4, MAZR, KROX, NKX6.1 and PXR (Fig. 2B). For glucocorticoid-repressed genes, binding motifs for GR and AR were still highly representative (Fig. 2C). In addition, HSF, STE11, PPAR, NaNog, MAZ, PAX, BR-C binding sites were significantly present (Fig. 2D). Comparing motifs identified between glucocorticoid-activated and repressed genes, four motifs GR BR-C, PAX and STE11 are present in both groups of genes. However, there are also motifs that are specifically represented in either glucocorticoid-activated or -repressed genes.

### Characterization of Glucocorticoid-regulated Genes Involved in TG Homeostasis

Gene ontology analysis showed that glucocorticoids regulate genes involved in distinct aspects of lipid metabolism (Dataset S4). In this list, several genes regulate TG homeostasis—*Scd-2*, *Vldlr*, *GPAT3* and *Lpin-1* (Dataset S4). *Cd36* has also been shown to play a role in fatty acid transport. Furthermore, ChIPseq identified several other genes involved in TG homeostasis, even though they were not regulated by 6-hour-DEX treatment in 3T3-L1 adipocytes. These genes include those involved in TG synthesis (*Scd-1*, *Scd-3*, *GPAT4*, *Agpat2*), lipolysis (*Lipe and Mgll*), lipid transport (*Lrp-1*, *Slc27a2*), and lipid storage (*S3-12*). It is possible that some of these genes are regulated at DEX-treatment-time points other than 6-hours. Alternatively, some of these genes may only be regulated in vivo, a condition that cannot be recapitulated by in vitro cell culture model, even though the GR binding phenotype is conserved in 3T3-L1 adipocytes. Overall, there are 14 genes involved in TG homeostasis in the gene list from ChIPseq, and we decided to further the studies in vivo, to determine whether these genes are regulated by glucocorticoids. The rationale in vivo studies is to allow us to monitor the in vivo metabolic effects of glucocorticoids in conjunction with gene expression analysis. Mice were treated with either 5 mg/kg DEX or equal volume of PBS for 4 days. The expressions of all 14 genes were significantly induced by DEX treatment in inguinal fat except for *Scd-3* (Table 2). In contrast, the effects of DEX on these genes in epididymal fat depot were minimal (data not shown).

**Table 2:** The Regulation of Genes Involved in TG Homeostasis by DEX.

		Fold Induction	SEM	P Value
TG Synthesis	*Scd1*	1.43	0.3	0.05
	*Scd2*	2.46	0.48	0.007
	*Scd3*	1.43	0.27	0.14
	*Gpat3*	1.98	0.20	0.001
	*Gpat4*	1.48	0.28	0.01
	*Agpat2*	1.77	0.22	0.003
	*Lipin1*	1.82	0.2	0.001
Lipolysis	*Lipe*	1.9	0.24	0.001
	*Mgll*	4.0	0.83	0.001
Lipid Transport	*Cd36*	1.68	0.17	0.006
	*Lrp1*	2.32	0.35	0.001
	*Vldlr*	2.07	0.37	0.02
	*Slc27a2*	3.17	1.27	0.01
Lipid Storage	*S3-12*	4.15	0.7	0.006

The fold induction of gene expression by DEX treatment (DEX/PBS) was the average of 10–12 mice used in each group.

**Figure 3:**
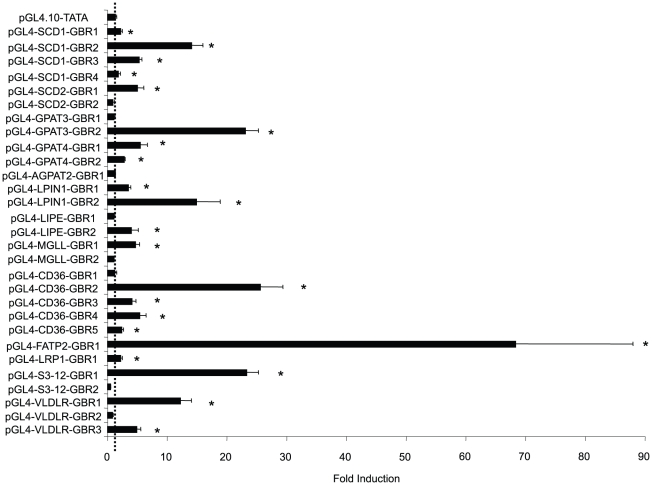
Glucocorticoid responsiveness of GBRs. Reporter plasmids that harbor distinct GBRs were co-transfected with pcDNA3-hGR (150 ng) and pRL (100 ng) into 3T3-L1 preadipocytes in a 24-well plate (*n* = 6 per group). pRL plasmid provided *Renilla* luciferase expression to document transfection efficiency. Transfected cells were left overnight, then washed with PBS and treated with 0.5 µM DEX for an additional 16–20 hrs. Cells were then lysed and assayed for firefly and *Renilla* luciferase activities. Data shows fold-induction of luciferase activity (DEX/ethanol treated samples) from at least 6 experiments (*, p<0.05). The error bars represent the S. E. for the fold induction. The dashed-line represents one fold.

The key criterion for a primary target gene is that its transcription is directly regulated by GR. Thus, its GBR(s) should contain functional GRE(s) that can mediate glucocorticoid response. To test whether GBRs from genes involved in TG homeostasis can confer hormonal response, each GBR was inserted in front of a TATA box of a heterologous reporter plasmid that drives a firefly luciferase gene (pGL4.10-TATA). Most of these genes contain or locate nearby multiple GBRs (Dataset S1). We subcloned each individual GB into the PGL4.10-TATA. The entire collection of reporter plasmids used in this report is listed in Table S1. These reporter plasmids were transfected into 3T3-L1 preadipocytes. 16–24 hours after transfection, cells were treated with either DEX or ethanol. After 24-hour-treatment with the hormone or ethanol, cells were lysed and luciferase activities were measured. We found that except for *Agpat2*, all other genes had at least one GBR that conferred hormonal response (Fig. 3). Overall, these results suggested that 12 of these 13 displayed genes were potential GR primary target genes.

### Glucocorticoids activated both TG synthesis and lipolysis in mouse inguinal fat pad

**Figure 4:**
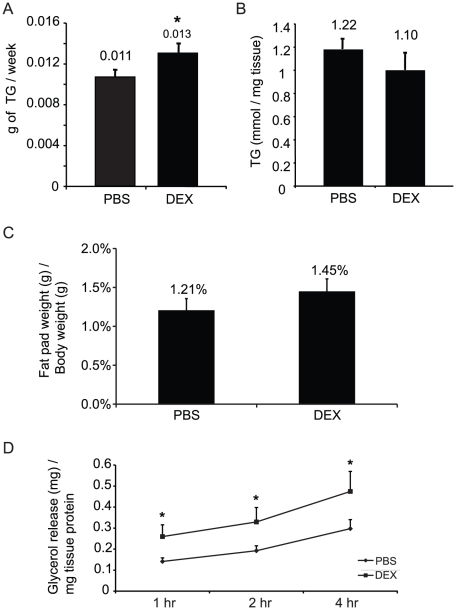
The glucocorticoid effect on TG metabolism. A). The effect of DEX on inguinal fat TG synthesis rate. The error bars represent the S. E. for the rate of TG synthesis (g of TG/week), with * representing a p value<0.05. B) Inguinal fat TG content measured by TLC from the same mice as in A). The error bars represent the S. E. for the TG concentration. C) Inguinal fat pad weight after the treatment of PBS or DEX for 4 days. The error bars represent the S. E. for the weight of inguinal fat pad. The p value for this experiment is 0.09. D) The effect of DEX on inguinal fat pad lipolysi. The error bars represent the S. E. for the glycerol release (mg) per mg tissue protein with the * representing a p value<0.05. 10–12 animals are used in all four experiments.

Our data showed that in the inguinal fat depot, 4-day DEX treatment concomitantly induced the expression of primary target genes encoding enzymes in TG biosynthesis and lipolysis, two opposing metabolic pathways. We therefore investigated whether DEX treatment affected both pathways in vivo. Stable isotope (heavy water) labeling technique was employed to label any newly synthesized TG-glycerol in the mice. The rate of TG synthesis can then be measured based on the percentage of deuterium incorporated into the TG-glycerol molecule. We found that TG synthesis rate in inguinal fat pad of DEX-treated animals was significantly higher than that of PBS-treated animals (Fig. 4A).

If glucocorticoids only increased the rate of TG synthesis, it would result in an elevated accumulation of TG levels in the inguinal fat pad. At the same time, if glucocorticoids also activated lipolytic pathways, the newly synthesized TG would be degraded instead of accumulated in adipose tissue. We proceeded to measure the TG levels in inguinal fat pad after 4-day-DEX or PBS treatment. Our results show that the TG levels were not significantly different between DEX- or PBS-treated animals (Fig. 4B). Furthermore, the weight of inguinal fat pad was not significantly different between DEX- and PBS-treated animals (Fig. 4C). Thus, these results indicated that 4-day-DEX treatment also activated lipolysis in inguinal fat pad. T further confirm that DEX induced lipolysis in inguinal fat, mice were treated with DEX for 4 days. At the end of treatment, inguinal fat pads were isolated and incubated in Krebs-Ringer Buffer. We then measured the glycerol levels in the buffer after 1, 2 and 4 hours. We found that the levels of glycerol released from DEX-treated inguinal fat pads were significantly higher than those of PBS-treated inguinal fat pads (Fig. 4D). Thus, these data demonstrated that DEX treatment for 4 days enhances lipolysis in inguinal fat depots. Overall, these in vivo results were in agreement with the fact that 4-day-DEX treatment induced genes encoding enzymes in both TG biosynthetic and lipolytic pathways.

### Increased expression of specific glucocorticoid-regulated genes in inguinal fat of mice overexpressing corticotropin-releasing hormone (CRH)

**Figure 5:**
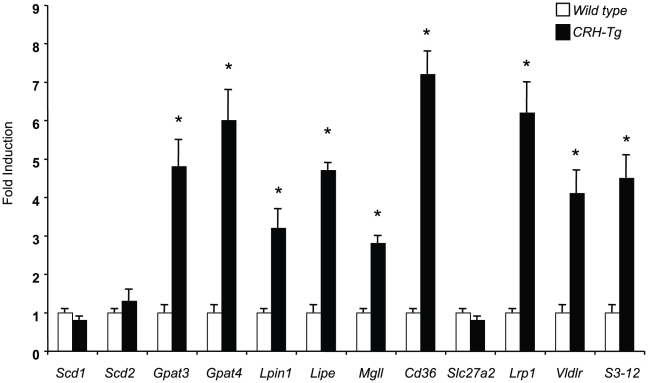
Comparing the expression of glucocorticoid-regulated genes in inguinal fat of CRH-Tg and wild type mice. Total RNA of inguinal fat of wild type and CRH-Tg mice were isolated and converted to cDNA. Real-time PCR was then performed to monitor the expression of genes indicated. Data shows fold-induction of gene expression (CRH-Tg/wild type) from 6–8 mice (*, p<0.05). The error bar represents the S.E. of the fold induction.

We next investigated whether the expression of these 12 glucocorticoid-regulated TG homeostasis-related genes was elevated in inguinal fat of mice overexpressing CRH (CRH-Tg) [23] CRH overexpression causes an increase of adrenocorticotropic hormone (ACTH) secretion from pituitary, which in turn elevates the secretion of corticosterone from adrenal gland. These mice, thus, have chronic high levels of corticosterone, which resemble human patients with Cushing's syndrome. Interestingly, the inguinal fat of these mice was found to have increased rates of both TG synthesis and lipolysis concomitantly (DJ Roohk and C Harris, personal communication). This phenotype is similar to mice injected with DEX for 4 days in our studies. We found that the expression of 9 of these 12 genes were significantly higher in the inguinal fat of CRH-Tg than in wild type mice (Fig. 5). These genes include those involved in both TG biosynthetic (*GPAT3*, *GPAT4* and *Lpin1*) and lipolytic (*Lipe* and *Mgll*) pathways. These results suggested that most of the potential GR primary targets identified in this study were involved in the regulation of TG metabolism in the adipose tissue upon chronic glucocorticoid treatment.

## Discussion

In this report, we provide three major findings. First, we present a list of GR primary target genes in adipocytes. Glucocorticoids have been shown to modulate the expression of certain genes in adipocytes previously, but it is unclear whether these genes are directl regulated by GR. Examples include *Scd-1* [52], *Vldlr* [53] and *Lipe* [54]. Here, we show that they are directly regulated by GR. These results and other novel GR primary genes identified from this study shine new light in the mechanisms of GR-regulated biological pathways in adipocytes. Second, we have identified genome-wide GBRs, which allows further elucidation and comparison of the mechanisms of GR-regulated transcription on distinct target genes in adipocytes. Finally, we show that, in adipose tissue, glucocorticoids are capable of increasing the rate of TG synthesis and lipolysis concurrently. We also identify the potential GR primary target genes involving in this process.

In our ChIPseq experiments in 3T3-L1 adipocytes, 8,848 GR binding sites are identified from the mouse genome. A similar range of number of binding sites were isolated using ChIPseq in other laboratories. Reddy et. al. identified 4,392 GR binding sites in human lung adenocarcinoma cells, A549 [33]. In human breast cancer cells, MCF7, 10,205 estrogen receptor binding sites were identified [32]. Furthermore, more than 5,000 peroxisome proliferator activated receptor gamma binding sites were isolated in murine 3T3-L1 cells [34] [55]. Steger et. al. recently identified 4,007 GR binding sites in 3T3-L1 preadipocytes [56]. Similar to our findings, these reports also showed that ∼10% of binding sites are located within 5 kb from the TSS. Conversely, significant numbers of binding sites are located in regions far distant from the TSS or introns. It is important to note that many of these intronic and long-range GR binding sites did mediate glucocorticoid response when placed in front of a TATA box in a heterologous reporter plasmid in our experiments. These results strongly suggest that they are functional GREs. Notably, GBRs located far away from the TSS are capable of interacting with basal transcription machinery [57]. Nonetheless, the configurations of these synthetic reporter constructs are not identical to their chromosomal context. Ultimately, disruption of an enhancer in the context of whole genome is needed to test its importance for the regulation of a given gene *in vivo*. Therefore, to study the transcriptional regulatory mechanisms of these binding sites in the natural chromosomal context will be a critical challenge for understanding GR action.

Among genes identified by ChIPseq, 337 genes found are significantly regulated by DEX in a microarray experiment. Notably, the analysis of gene expression using microarray could miss specific splicing variants of certain genes; it is likely that more genes are regulated by glucocorticoids in this ChIPseq list. Some of these genes have been previously shown to participate in the regulation of insulin signaling and adipogenesis. However, whether these sets of target genes can mediate the glucocorticoid response will require further study. From our gene ontology analysis, genes involved in angiogenesis and blood vessel development are significantly represented. As angiogenesis plays a role in the expansion of adipose tissues, it would be interesting to investigate whether glucocorticoids affect this process in vivo in future experiments. It is important to note that the biological functions of many of these 337 genes in adipocytes have not been studied. The identification of their biological functions provides new insights for glucocorticoid biology in adipocytes.

Consensus motif analyses suggest that predicated GRE sequences are located in or nearby most glucocorticoid-activated genes. Interestingly, the predicated GRE motif is also highly represented in GBRs of glucocorticoid-repressed genes. Whether these predicated GRE motifs mediate the repressive effect of GR will be an important topic in our future studies. GR frequently represses the transcription through tethering GREs, at which GR does not bind to DNA directly; instead it is recruited to GREs through interaction with other transcription factors. Two most common tethering GREs are NFκB and AP-1 response elements [58]. However, in our bioinformatics analyses, these two binding motifs are not among the most representative sequences in GBRs of glucocorticoid-repressed genes. Instead, the motifs identified have not been linked to the repressive effect of GR (Fig. 2D). C/EBP and HNF3alpha, two families of transcription factors whose binding motifs are highly represented in GBRs of glucocorticoid-activated genes, were previously shown to act with GR to participate in glucocorticoid-activate gene transcription [59], [60], [61], [62]. The HNF3alpha binding motif is similar to those of the FOX family transcription factors. HNF3alpha and FOX are also represented in GBRs of glucocorticoid-induced genes. Notably, the interaction between GR and other transcription factor binding motifs identified in GBRs of glucocorticoid-activated genes has not been reported. Overall, these analyses identify potential novel interactions between GR and other transcription factors in adipocytes. We shall further investigate the biological relevance of these possible interactions in future experiments.

Glucocorticoids appear to regulate distinct aspects of lipid metabolism (Dataset S4). In this report, we focus on their effects on TG homeostasis. We are the first to show that *Scd-1*, *2*, *GPAT3*, *GPAT4* (TG synthesis), *Lipe*, *Mgll* (lipolysis), *Cd36*, *Vldlr*, *Lrp-1*, *Slc27a2* (lipid transport), *S3-12* (lipid storage) are likely directly regulated by GR based on the fact that their expressions are regulated by glucocorticoids and they have functional GBRs. Although *Scd-1* [52], *Vldlr* [53] and *Lipe* [54] have previously been shown to be regulated by glucocorticoids in 3T3-L1 cells or primary adipocytes, the location of their GREs were not reported until now. Involved in TG synthesis, *Lpin1* was previously shown as a GR primary target. The GRE identified is located between −311 and −297 of *Lpin1* promoter (relative to TSS, whose chromosomal location is chr12: 16615250 based on mm8 assembly or chr12: 16597172 based on mm9 assembly) [63]. In our ChIPseq experiment, we identified a GBR that is approximately 200–400 bp away from this GRE (Dataset S1, row 1474). It is possible that this GBR is from the recruitment of GR to previously identified GRE. In addition, we found two GBRs that are located ∼1 kb apart between −26,920 and −27,250 (LPIN1-GBR1) and −27,980 and −29,022 (LPIN1-GBR2) of the *Lpin1* gene. Both GBRs identified in our studies were highly responsive to DEX in reporter assays, especially LPIN1-GBR2 (Fig. 3). Thus, it is possible that *Lpin1* is regulated by multiple GREs. Notably, several genes in this list are found regulated by glucocorticoids in vivo but not in 3T3-L1 adipocytes. In fact, *Lipe* was found induced by glucocorticoids in rat primary adipocytes, but its expression was not activated by glucocorticoids in our experiments. There are several possible explanations for these observations. It is possible that the regulation of these genes by glucocorticoids require specific transcriptional factors or cofactors that are expressed at low levels in 3T3-L1 cells. Another possibility is that the regulation of these genes by glucocorticoids may require the assistance of other signals, which are not active in 3T3-L1 adipocytes. We are currently investigating the regulation of these genes in primary adipocytes to further understand how glucocorticoids regulate these genes.

Interestingly, two 1-acylglycerol-3-phosphate O-acyltransferases (*Agpats*) contain GBRs in their genomic regions: *Agpat3* [64] and *Agpat4* [65] (Dataset S1). We find that the expression of *Agpat3* and *4* is not affected by 4-day DEX treatment in inguinal fat (data not shown). However, *Agpat4* expression is induced in 6-hour-DEX treatment in 3T3-L1 adipocytes and 24-hour-DEX treatment in mice, and *Agpat3* expression is elevated in inguinal fat of CRH-Tg mice (data not shown). Interestingly, the GBRs of *Agpat4* are able to mediate glucocorticoid response when inserted into a reporter plasmid (data not shown). Thus, *Agpat4* is a potential GR primary target. Our ChIPseq also located a GBR in the *Angptl4* gene (Dataset S1), which is involved in adipose tissue lipolysis [66]. This GBR is located downstream of the stop codon of mouse *Angptl4* gene, in a similar location to the GRE we previously identified in rat *Angptl4* gene [67]. As we previously presented, *Angptl4* gene is not regulated by glucocorticoids in 3T3-L1 adipocytes, but its expression is induced by DEX treatment in epididymal fat pad [67]. Overall, the identification of these novel GBRs in these genes involved in TG homeostasis should facilitate future studies on how glucocorticoids regulate their transcription and TG metabolism.

Notably, genes involved in lipid metabolism were not enriched in GBRs identified in 3T3-L1 preadipocytes. We compared GBRs identified in our studies and 3T3-L1 preadipocytes [56], and found that 1,804 binding sites are common (Dataset S4). In 337 of the glucocorticoid responsive gene identified in 3T3-L1 adipocytes, 153 genes have GBRs in this list of 1,804 overlapping binding sites (Dataset S5). When we compared the list of 29 genes involved in lipid metabolism in adipocytes with these 153 genes, we found 11 genes were overlapped (Dataset S5). Among 12 glucocorticoid responsive genes involved in TG homeostasis, the GBRs for *Gpat3*, *S3-12*, *Mgll*, *Lipe*, *Lrp1*, and *Slc27a2* were not found in 3T3-L1 preadipocytes ChIPseq. As the biological role of glucocorticoids somewhat differs in preadipocytes and adipocytes (differentiation vs. metabolism), it is not a surprise that GR regulates distinct sets of genes in these two cell types. More detailed analyses of transcriptional regulatory mechanism of each of these genes will be required to learn the mechanism governing this differential regulation. Interestingly, a recent study showed the distinct genome-wide chromatin marks between preadipocytes and adipocytes [68]. This may affect the recruitment of GR to certain GBRs in genome.

Applying stable isotope labeling technique, we find that the rate of TG synthesis in the inguinal fat pad of 4-day DEX-treated animals is higher than that of PBS-treated animals. However, the levels of TG in inguinal fat pad are not significantly different between DEX- and PBS-treated animals. This indicates the activation of lipolysis, which is in agreement with the fact that DEX treatment for 4 days activates the genes encoding enzymes of both TG synthesis and lipolysis. Although previous studies have independently shown that glucocorticoids either stimulate lipolysis or TG synthesis (19), our results are first to show that glucocorticoids activate both pathways in the same fat depot. Thus, glucocorticoids appear to cause futile cycling in inguinal fat, in which TG was continuously synthesized and degraded in our experimental condition. These newly generated fatty acids could have three routes. First, fatty acids can be re-incorporated into TG in adipocytes. Second, these fatty acids could re-distribute to liver and skeletal muscle, where they were re-synthesized to TG. We have previously observed the increased TG levels in liver upon 4-day DEX treatment [67], and an increase level of TG in skeletal muscle has been previously reported [69]. Finally, fatty acids could also be oxidized. It is unclear why glucocorticoids induce this type of futile cycling in the adipose tissue. It is possible that during the stress condition, glucose needs to be preserved, and the role of glucocorticoids in adipose tissue is to provide fatty acids to plasma, so other tissues can use them as energy sources.

Importantly, the expression of most GR responsive genes identified in this study is also increased in CRH-Tg mice with a chronic elevation of plasma corticosterone levels. The lipid phenotypes of these mice are somewhat similar to the mice treated with DEX for 4 days in our study (Donald J. Rhook and Charlie Harris, personal communication), as the rates of TG synthesis and lipolysis are induced at the same time in the inguinal fats of both types of mice. These results suggest that most genes identified from this study also participate in the long-term glucocorticoid effect on TG metabolism. In human, chronic glucocorticoid treatment causes lipid re-distribution between distinct fat depots, it is possible that longer treatment of glucocorticoids leads to distinct local environments at different fat depots, at which specific hormonal and/or autocrine/paracrine factors act together with glucocorticoids to differentially regulate the TG biosynthetic or lipolytic primary target genes identified in this report. Future studies should investigate this model.

## Supporting Information

Dataset S1 **GBRs identified from ChIPseq.** GBRs were identified using MACS as described in Methods. The distribution of GR-binding sites, relative to nearest genes, was determined with PinkThing (http://pinkthing.cmbi.ru.nl). All sequences associated with the peaks were obtained from the *Mus musculus* NCBI m37 genome assembly (mm9; July 2007).
(XLS)

Dataset S2 **Genes regulated by DEX in 3T3-L1 adipocytes.** Microarray was performed as described in Methods. Genes that are induced or repressed by DEX treatment for more than 1.5 fold with p value<0.05 are listed.
(XLS)

Dataset S3 **GR-regulated Genes containing or locating nearby GBRs in 3T3-L1 adipocytes.** The overlapping genes identified in both ChIPseq (Dataset S1) and microarray (Dataset S2) were isolated by a web-based bioinformatic tool, Venn Diagram Generator (http://www.pangloss.com/seidel/Protocols/venn.cgi). DEX-activated or repressed genes are shown separately.
(XLS)

Dataset S4 **Gene Ontology Analysis of Glucocorticoid Responsive Genes.** Glucocorticoid responsive genes analyzed using gene ontology software (described in Methods). The genes identified in top categories of the functional annotation chart are shown.
(XLS)

Dataset S5 **Overlapping GBRs identified in 3T3L1 adipocytes and preadipocytes.** The overlapping 1,804 GR binding regions in 3T3-L1 preadipocytes (56) and adipocytes (this report) were shown. Furthermore, genes that locate nearest to these 1,804 GR-binding regions are shown.
(XLS)

Table S1 **Reporter Assays of GBRs.**
(XLS)

Table S2 **Primers used for cloning GBRs into pGL4.10E4TATA reporter plasmids.**
(XLS)
